# The Importance of Bushmeat in the Livelihoods of West African Cash-Crop Farmers Living in a Faunally-Depleted Landscape

**DOI:** 10.1371/journal.pone.0072807

**Published:** 2013-08-16

**Authors:** Björn Schulte-Herbrüggen, Guy Cowlishaw, Katherine Homewood, J. Marcus Rowcliffe

**Affiliations:** 1 Institute of Zoology, Zoological Society of London, London, United Kingdom; 2 Department of Anthropology, University College London, London, United Kingdom; Université de Strasbourg, France

## Abstract

Bushmeat is an important resource in the livelihoods of many rural communities in sub-Saharan Africa and may be a crucial safety-net for the most vulnerable households, especially during times of economic hardship. However, little is known about the impacts of wildlife depletion on these functions. This study quantifies the role of bushmeat in diversified rural household economies in a wildlife depleted forest-farm landscape in Ghana, assessing its importance overall, as well as differentiated by the relative vulnerability of households. Using repeat socioeconomic questionnaires (N=787) among 63 households over a one-year period, the following hypotheses were tested: (a) vulnerable households harvest more bushmeat; (b) bushmeat contributes a greater proportion of household production in vulnerable households; (c) bushmeat is more important for cash income than consumption in vulnerable households; and (d) bushmeat sales are more important for vulnerable households. The bushmeat harvest value averaged less than US$1.0 per day for 89% of households and comprised less than 7% of household production value. Household wealth and gender of the household head had little effect on the importance of bushmeat. However, bushmeat harvest and sales were highest during the agricultural lean season. Overall, most harvested bushmeat (64%) was consumed, enabling households to spend 30% less on meat/fish purchases. These findings suggest that, despite heavily depleted wildlife and diversified livelihoods, bushmeat continues to have an important role in rural livelihoods by acting as a safety net for income smoothing and reducing household expenditure during times of economic hardship.

## Introduction

There is growing awareness of the importance of ecosystem services such as the harvest of non-timber forest products (NTFP) for rural communities in developing countries [1–3]. An estimated 1.6 billion people depend partly or fully on forest products to sustain their livelihoods [4]. Where income-generating livelihood options are scarce, the sale of NTFPs is often the only means to earn a cash income [5]. This suggests a link between NTFP harvest and human wellbeing, which has recently gained increasing attention in conservation, development and policy circles [6] and among funding bodies [7].

Bushmeat is an important NTFP throughout sub-Saharan Africa, worth millions of dollars in trade [8]. It has many properties favourable to commercialisation, such as high price-to-volume ratio and flexible allocation of labour inputs [9]. Hunters supplying bushmeat to traders may exert strong bargaining power within the rural-urban commodity chain [10] and can gain incomes comparable to or higher than average local wages [11–14]. This suggests a potential role for bushmeat in contributing to human wellbeing and poverty alleviation.

However, the sustained importance of bushmeat for rural livelihoods is questionable for two reasons. Firstly, current bushmeat harvest levels are unsustainable and wildlife populations are declining throughout the tropics [15–17]. Estimated sustainable offtake levels are pegged far below current harvest levels in African forests [15] and it is not clear whether a sustainable harvest would generate sufficient income to lift people out of poverty [18]. Secondly, evidence about the importance of bushmeat in rural livelihoods is primarily derived from studies conducted in environments with abundant wildlife and with few alternative opportunities for earning income. For example, a recent study in rural Equatorial Guinea showed that bushmeat is the only source of cash income for 59% of men in the village [19]. This predominance of a single income source contrasts strongly with other sub-Saharan livelihoods studies, which show that income diversification is widespread, and that the importance of farm income and nonfarm income, of which bushmeat is a part, varies greatly across localities [20]. Hence wildlife depletion and increasing opportunity costs of bushmeat hunting due to a household’s engagement in alternative income generating activities [21] may reduce the importance of bushmeat as a source of cash income and consequently its contribution to human wellbeing and poverty alleviation.

On the other hand, the open access nature of bushmeat harvesting systems, the relatively low entry costs compared to other activities, and the flexible timing of hunting effort allocation, mean that bushmeat may be important to disadvantaged households, such as the rural poor or female-headed households (hereafter FHH) [22–25], and to a wider cross-section of households during the agricultural lean season. The seasonality of tropical farming systems results in temporal fluctuations in income and production flows, thereby exposing households to income and consumption shortages [26]. This is especially the case for poor and FHHs, which hold limited capital assets and are restricted in their options for diversifying income and production sources [27]. Bushmeat has been shown to be of pivotal importance at times of acute shortage in the livelihoods of the most vulnerable by helping to overcome shortages of food [28,29] and income [30,31].

Few bushmeat studies have been conducted among diversified farming households living within a faunally-depleted environment [32] and their conclusiveness regarding the importance of bushmeat, especially for vulnerable households, is hampered by small sample sizes and/or short data collection periods, as well as a lack of focus on household vulnerability. For example, Dei’s study [28] of cash-crop farmers living in a wildlife-depleted environment in Ghana was limited to 20 households. At the time, he concluded that bushmeat was important for consumption, especially for poor households during the lean season, although it was not an important source of income. Another study conducted recently in a nearby area sampled a larger number of households (388) during a brief period of two months during the lean season, and concluded that bushmeat was a major source of household income, providing 35% of total village income, compared to 25% from farming [33]. In contrast, [34] reported that Southeast Asian households living in wildlife depleted forests did not depend on bushmeat for consumption, “because the resource is simply not there any more“.

It is difficult to draw conclusions from studies that vary in their depth of data collection, at sites with varying degrees of wildlife depletion and access to alternative income sources, conducted at different times over the last few decades. However, it nevertheless appears that depleted wildlife populations continue to support rural livelihoods, albeit to a lesser extent than where wildlife populations are abundant [35]. Where attempts have been made to record socio-economic household characteristics [28], it seems that the importance of bushmeat continues to be higher in chronically poor households, and in households with temporarily low income. However, overall the existing information about the importance of bushmeat to vulnerable households, particularly during the agricultural lean season, is limited. This hampers the development of policy and management interventions to support rural livelihoods and conservation [36].

This study aims to improve our understanding of the potential for bushmeat harvested from depleted wildlife populations to support vulnerable households, especially at times of economic hardship. The study was based among Ghanaian cocoa farmers with access to a diverse range of income and production sources, and living within a forest-farm landscape with depleted wildlife. The study investigated the effects of household vulnerability on the importance of bushmeat, where vulnerability was defined in terms of poverty, being female-headed or during the agricultural lean season. We tested four hypotheses: (1) vulnerable households harvest more bushmeat; (2) bushmeat contributes a greater proportion to household production of vulnerable households; (3) bushmeat is more important for cash income than consumption in vulnerable households; and (4) bushmeat sales are more important for vulnerable households.

## Methods

### Study site

**Figure 1:**
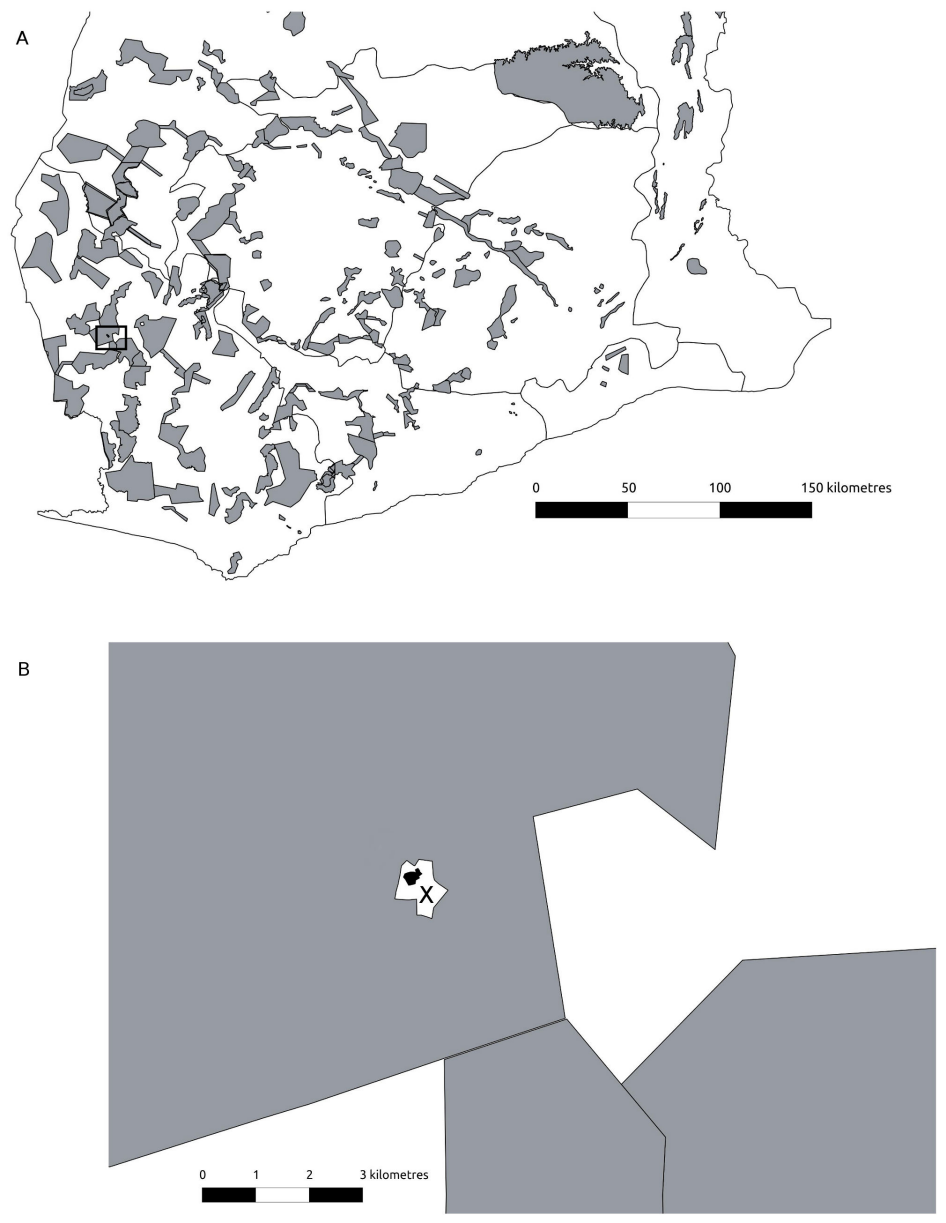
Map of the study area showing: A) southern Ghana with forest reserves and protected areas (grey). B) Wansampo with the village (black) being surrounded by farmland (white with cross) inside the Sui Forest Reserve.

This study was carried out in the village of Wansampobreampa (hereafter Wansampo) (6.06N, -2.73W) in the Akontombra district, Western Region, SW Ghana (Figure 1). The community has about 350 people living in 70 households. The village is bisected by a laterite road that connects two district capitals (Sefwi Wiawso and Akontombra) and is accessible all year. Frequent traffic of passenger cars facilitates transportation to district markets. The road developed out of a feeder road that was built by a timber company during the 1960s.

Wansampo is a cocoa farming community located inside the Sui River Forest Reserve (FR) that is managed for timber production. Farmland is intensively cultivated, predominantly for cocoa production. Some farmland occurs inside the Reserve, but the majority lies outside it. Cocoa was planted on 59% of farms and it was the dominant crop on 45% of farms. The vast majority of cocoa farms (81%) were monocultures, with very few or no crops planted under the cocoa trees. The remaining cocoa farms that still had a large amount of food crops were in the process of becoming monocultures. The agricultural calender is split into three periods: the cocoa season (October to January), during which the frequency and value of cocoa sales peaks; the post-cocoa season (February to June), when the sales frequency and value declines; and the lean season (July to September), when the sales frequency and value are at their lowest.

### Ethics statement

The research was carried out in accordance with the ethics guidelines of the Association of Social Anthropologists of the UK and Commonwealth (ASA) and the methods were approved by the Department of Anthropology Ethics Committee, University College, London, prior to data collection. A meeting was also held with all community members prior to data collection to carefully explain the purpose of the study and to obtain informed consent of the research participants. Because of the low level of literacy in the area it was not possible to obtain written consent. All data were anonymised to reduce the risk of harm to informants. The research was associated with a project of the Zoological Society of London operating in a nearby area and was covered by its research permits obtained from relevant authorities.

### Data collection

The study took place over a period of twelve months (July 2008 to June 2009) including the full agricultural cycle comprising the main agricultural harvest season and the lean season. Prior to the survey period, Björn Schulte-Herbrüggen spent six months in the village piloting the questionnaire, familiarising himself with local livelihoods and establishing relationships with the villagers to ensure high data quality (for a discussion of this subject see 37). All interviews were conducted in Twi/Sefwi, by BSH or a volunteer from the USA, with help from local assistants. All assistants received extensive training in social research methods prior to data collection. The mean currency exchange rate was US$1.0 = 11,862 Cedis (June 2008 to June 2009; http://www.oanda.com/).

### Household demography and wealth

Household demographic information was collected during a census in August 2007 and revised during two further censuses (April 2008 and June 2009), recording information on the household head, number of household members, their age and education. Repeated assessments were required since household composition varied strongly throughout the data collection period and was in many cases ambiguous during the first and second censuses. Data from the third census benefited from the prolonged observation period in the village, particularly from dinner surveys recording household members present, providing an in-depth understanding of relationships between households and dependencies. Household demographic data included in the models were derived from the final census, as it was considered of the highest quality.

Participatory household wealth ranking exercises were conducted with seven long-standing community members of different genders, socio-economic backgrounds and community neighbourhoods. All households were grouped into four wealth categories based on their mean participatory wealth score (following [38]). To calculate mean wealth scores, each household was assigned a value during each of seven ranking exercises, depending on the group it was allocated to and on the total number of wealth groups chosen during that exercise. This was repeated for every exercise and the mean estimated across exercises. The cut-off points between wealth groups were calculated by subtracting the highest mean wealth score obtained by any household from the mean lowest score obtained and dividing the result by three. This provides three cut-off points to obtain four wealth groups. Outcomes of the wealth ranking were cross-checked against independent wealth characteristics (household expenditure and house roof value) and were strongly correlated [39].

### Socioeconomic household surveys

Semi-structured questionnaires were used to assess the harvest and use of bushmeat, household production and expenditure, and the consumption of meat and fish. A total of 787 complete interviews were used in this study, covering 63 households.

Information on bushmeat harvest and use (consumption, sale or gift) was collected using 24hr and two-week recalls. Hunters had little difficulty remembering bushmeat harvests during either period. However, eliciting reliable information about bushmeat use for the preceding two weeks was at times challenging. The analysis of bushmeat use data was therefore limited to 24hr recall data. Bushmeat was defined broadly, including wild mammals, birds, crabs, snails and tortoises. If a harvest event had taken place, the recorded data included the hunter ID, species harvested, number of animals harvested and sales price in the village. Interviewees commonly bought and sold agricultural products and bushmeat in the village and were familiar with local prices. For each harvest event within the last 24 hours, interviewees were further questioned as to whether the harvest was for consumption by household members, or sale or gift to non-household members. We did not differentiate between past and planned use, e.g. whether an animal harvested within the last 24 hours had already been consumed or would be consumed.

Estimates of the value of total household production (including farm produce and non-timber forest products) within 24 hours prior to an interview were obtained by eliciting the value of harvests that either (a) arrived at the house; (b) was consumed on the farm; (c) had already been sold; or (d) had already been given away as a gift. All produce was valued by interviewees in local sales prices. To avoid underrepresentation of large but infrequent cocoa harvests, these were recorded using two-week recall and the value divided by 14 to obtain daily production estimates.

Interviewees were asked to state all monetary expenditure incurred within the last 24 hours. To elicit infrequent large expenditures, two-week recalls were employed for single expenditures exceeding US$4.22 (50,000 Cedis). The threshold was judged appropriate for large infrequent expenditures and was confirmed as such by informants. For all expenditures the proportion of value used for consumption, sale and gifts was recorded.

Meat/fish consumption surveys elicited the type and monetary value of meat/fish consumed by household members for each meal (breakfast/lunch/dinner) or snack within the last 24 hours. These data were then cross-checked with data on wildlife harvest, meat/fish expenditure and gifts received for the same period. If mismatches were observed, these were discussed with interviewees. For every record of meat/fish consumed by household members it was also recorded whether it entered the household through purchase, own production (e.g. harvest of bushmeat), or as a gift. The majority of purchased meat/fish consumed was bought from traders in the village on a daily basis, facilitating the recall of expenditures by interviewees.

### Data analysis

All statistical analyses were performed in the R environment, version 2.9.2 [40]. To explore the relationship between a response variable and independent variables, Generalized Linear Mixed Models (GLMM) were used (’lme4’ package, version 0.999375-32 [41]), including household as a random effect in all cases.

The analysis focused on five household response variables: bushmeat harvest, contribution of bushmeat to household production, consumption and sale of harvested bushmeat and the consumption of meat/fish. The independent variables assessed during the analyses were our three measures of household vulnerability, namely participatory household wealth (4 factor levels), agricultural seasonality (3 factor levels: cocoa season/post-cocoa season/lean season), and gender of the household head (2 factor levels: female (FHH)/male (MHH)), plus a fourth variable, the consumption of harvested bushmeat (2 factor levels: yes/no).

To control for the confounding effects of household demographics and composition, four additional variables were included as fixed effects in the models: number of active (aged 16 to 65 years) male household members (continuous variable, range: 0 - 4); household dependence ratio (ratio of dependent to total number of household members, range: 0 - 0.75 in four factor level with thresholds at >0.2, >0.4 and >0.6); age of the household head (range: 21-84 years in six factor levels with thresholds at >30, >40, >50, >60 and >70); and household head level of education (number of years in formal education; range: 0-12 years in five factor levels with thresholds at >0, >3, >6 and >9). To assess whether bushmeat harvest reduced the need to purchase meat/fish, consumption of harvested bushmeat at the previous night’s dinner was related to the consumption of purchased meat during the same meal.

All models tested for interactions (“:”) and additive (“+”) effects (“*” abbreviates a combination of interaction and additive effects) between household wealth, season and gender of the household head (if these were part of the model). However, it was not possible to test for an interaction between household wealth and gender of the household head because there were no FHH in the highest wealth group. Similarly, it was not possible to assess the interaction between household wealth and season because the wealthiest households always consumed all harvested bushmeat and households from all wealth categories except the poorest always consumed all harvested bushmeat during the cocoa season.

Model evaluation was based on the information-theoretic approach using Akaike’s Information Criterion (AIC) to infer the relative support for alternative models [42]. The interpretation of GLMM results was based on two criteria. First, the _i_th model’s relative support was evaluated with reference to the model with the lowest AIC value using the AIC difference: ΔAIC_i_ = AIC_i_ -AIC_min_. Models with ΔAIC_i_ ≤ 2 were deemed to have substantial support, those with 4≤ ΔAIC_i_ ≤7 considerably less support, and ΔAIC_i_>7 essentially no support [43]. Secondly, the ΔAIC of the null model (hereafter ΔAIC_N_) provided a measure of the relative confidence in the interpretation of the results. If support for ΔAIC_N_ was high (≤ 2), then confidence in the alternative models was reduced, even if the best model was not the null. All interpretations of relative support for individual variables were further triangulated by assessing the respective effect sizes and standard errors and the ΔAIC_i_ of the univariate model. The validity of models regarding the assumed normal distribution of within-group errors and randomly distributed random effects were tested qualitatively by plotting within-group residuals, and inspection of fitted versus residual plots, respectively [44].

## Results

On days when bushmeat was harvested, the average value was US$3.2/household/day (±US$4.1 SD), ranging from US$0.04/household/day for a single crab or snail to US$25.3/household/day for the harvest of a large African civet ( *Civettictis* *civetta* ). However, across the whole survey period, harvest value averaged US$0.4/household/day (±US$1.75 SD). Only 5% of households harvested bushmeat with a mean daily value higher than the national minimum salary (US$2.24/day) and a further 5% of households harvested bushmeat worth more than half the minimum salary.

### Bushmeat harvest

**Figure 2:**
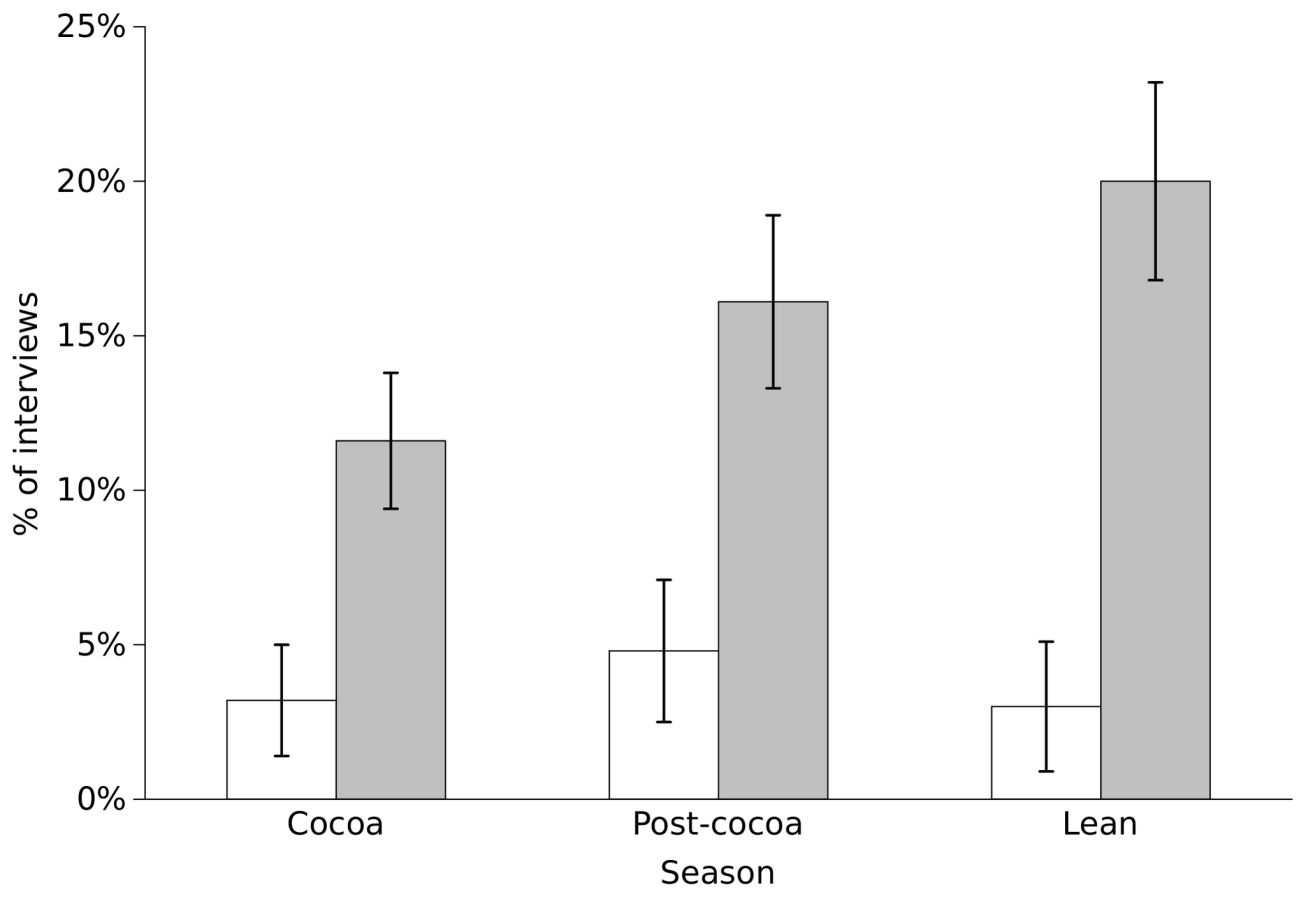
Seasonal variation in bushmeat harvest across female- and male-headed households. The percentage of interviews recording bushmeat harvest within 24 hours prior to interviews across the three agricultural seasons (cocoa, post-cocoa, lean season) for female- (white) and male-headed households (grey). Total sample size is 787 interviews across 63 households. Means and standard errors across households are shown.

To assess the relationship between vulnerability and bushmeat harvest, the evidence for an effect of income seasonality, household wealth and gender of the household head on both the likelihood of harvesting bushmeat and the value of the bushmeat harvest were examined. There was no evidence to suggest that the value of bushmeat harvested per day was affected by any vulnerability indicators (ΔAIC_N_ = 0, N=97). However, there was good support for the likelihood of bushmeat harvest being affected by income seasonality and gender of the household head, although not by household wealth (Table 1). Bushmeat harvest was most likely during the lean and post-cocoa season and least likely during the cocoa season, while male-headed households (MHHs) were on average three times more likely to harvest bushmeat than the more vulnerable FHHs (Figure 2). Furthermore, while FHHs showed no discernible difference in their bushmeat harvest likelihood among seasons, MHHs were less likely to harvest bushmeat during the cocoa season than during the lean season.

**Table 1:** Results of binomial GLMM analysis testing the effects of household wealth, seasonality and gender of the household head (HH) on the likelihood of a household harvesting bushmeat within 24 hours prior to an interview (N=787; No. households=63).

**Model**	**ΔAIC_i_**	**Akaike weight**
Season + HH	0	0.46
HH	0.8	0.31
Season * HH	3.2	0.09
Wealth + Season + HH	3.8	0.07
Wealth + HH	4.6	0.05
Wealth + Season * HH	7.1	0.01
Wealth * Season + HH	9.9	<0.01
Season	11.2	<0.01
Null model	12.1	<0.01
Wealth * Season + Season * HH	13.3	<0.01
Wealth + Season	16.5	<0.01
Wealth	17.4	<0.01
Wealth * Season	22.4	<0.01

### Importance of bushmeat in household production

On days when bushmeat was harvested, it comprised a mean of 44% of household production value (median=34%; range=0.5%-100%) but due to the relatively low frequency of harvesting, bushmeat comprised a mean of only 7% of total household production (median=0%; range=0%-100%) across the year. Given this relatively low contribution to total household production and the lack of a seasonal pattern in the value of bushmeat harvest, there was only marginal evidence for bushmeat to contribute more to household production during the lean season than during the cocoa season, and basically no evidence that bushmeat comprised a larger proportion of household production for poorer households or FHHs, nor was bushmeat more important to FHHs during the lean season (Table 2).

**Table 2:** Results of GLMM analysis assessing the proportion of household production value derived from bushmeat harvest in relation to household wealth, seasonality and gender of the household head (HH) (N=97; No. households=38).

**Model**	**ΔAIC_i_**	**Akaike weight**
Season	0	0.30
Wealth + Season	0.4	0.24
Null model	1.8	0.12
Season + HH	2.5	0.08
Wealth + Season + HH	2.7	0.08
HH	3.7	0.05
Wealth	3.9	0.04
Wealth + Season * HH	4.3	0.03
Season * HH	4.7	0.03
Wealth + HH	5.7	0.02
Wealth * Season	9.2	<0.01
Wealth * Season + HH	12.0	<0.01
Wealth * Season + Season * HH	13.1	<0.01

### Use of harvested bushmeat

Most harvested bushmeat was consumed within the hunter’s household. Interviewees who harvested bushmeat within 24 hours prior to an interview reported the consumption of all or part of the harvest in 93% of interviews, sale in 19% of interviews and gift in 13% of interviews, suggesting that even when bushmeat was sold or given away as a gift, some was usually kept for their own consumption. Of the total bushmeat value harvested, 64% was consumed, 26% was sold and 10% was given away as gifts.

Considering the high prevalence of bushmeat consumption, it is perhaps not surprising that neither seasonality nor household wealth nor gender of the household head affected the likelihood of consuming harvested bushmeat (ΔAIC_N_= 0, N=97). Similarly, there was no evidence for an effect on the value of bushmeat consumption on days with bushmeat harvest (ΔAIC_N_=0; N=86).

As harvested bushmeat was mainly consumed and there was no evidence for bushmeat consumption being more important for poorer households or FHHs or during the lean season, we hypothesised that the consumption of harvested bushmeat reduced household meat expenditure. Households consumed meat/fish on a nearly daily basis (92% of interviews) and spent on average US$0.92/day (SD=1.41) on buying meat/fish. This was equivalent to 42% of daily food expenditure and 29% of daily total household expenditure. The evening meal was the main meal during the day and of the total meat value consumed, 91% was consumed at this time.

**Figure 3:**
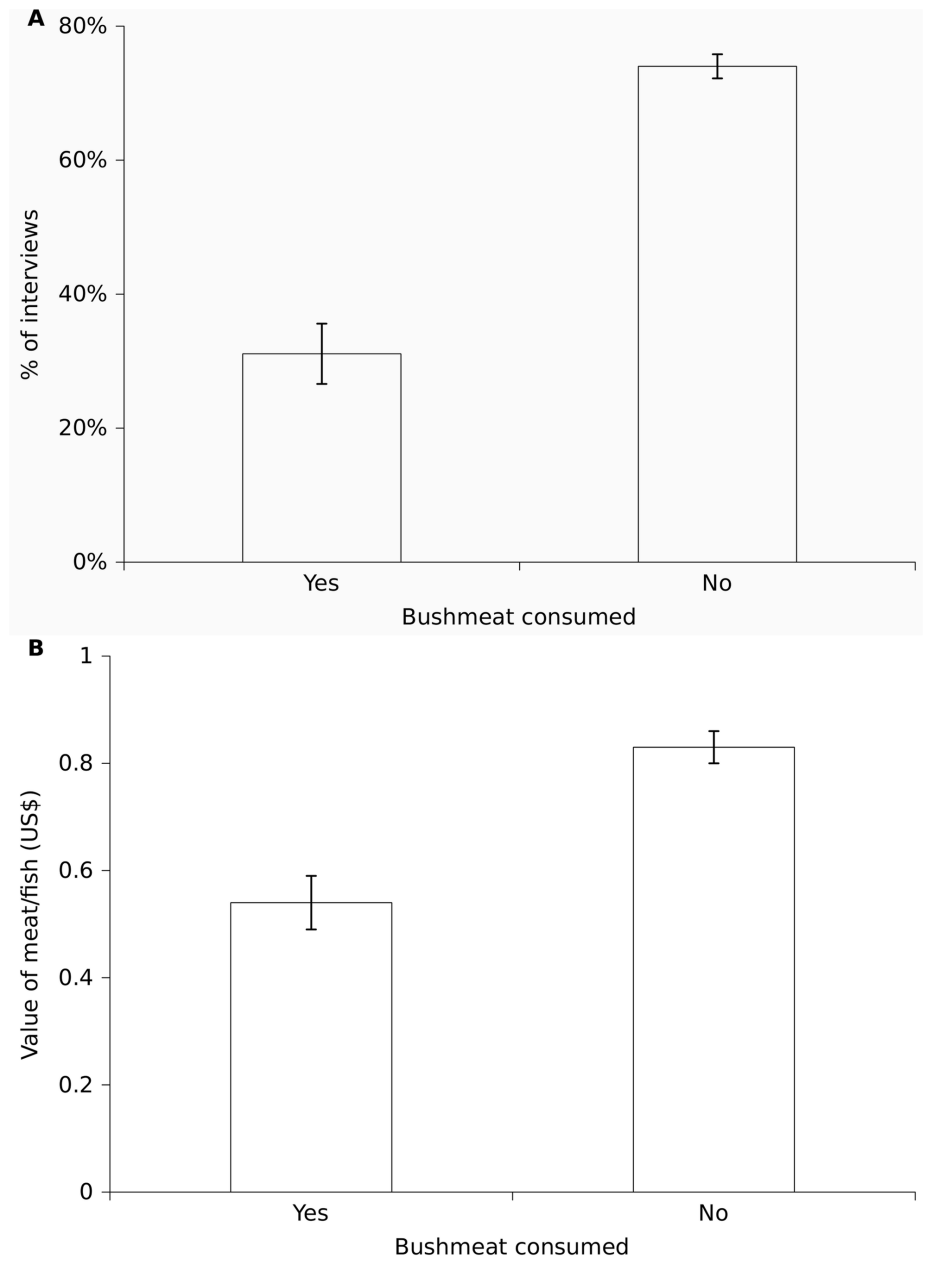
Effect of bushmeat consumption on the consumption of purchased meat/fish. Consumption of purchased meat/fish by household members at previous night’s dinner in relation to whether harvested bushmeat was consumed: (A) percentage of interviews reporting the consumption of purchased meat/fish; (B) value of purchased meat/fish. Means and standard errors are shown.

The consumption of harvested bushmeat reduced both the likelihood of consuming purchased meat/fish (ΔAIC_N_=64; N=694) and the value of purchased meat/fish consumption when it occurred (ΔAIC_N_=7; N=468), suggesting that bushmeat harvest resulted in savings to the household. When households consumed harvested bushmeat, only 31% of interviews recorded additional consumption of purchased meat/fish (Figure 3a). In contrast, when no bushmeat was harvested, 74% of interviews recorded purchased meat/fish for dinner, with the remaining 26% consuming meat/fish gifts. Furthermore, on days when purchased meat/fish was consumed, additional consumption of harvested bushmeat reduced the value of meat/fish consumed by about 30% (Figure 3b).

**Figure 4:**
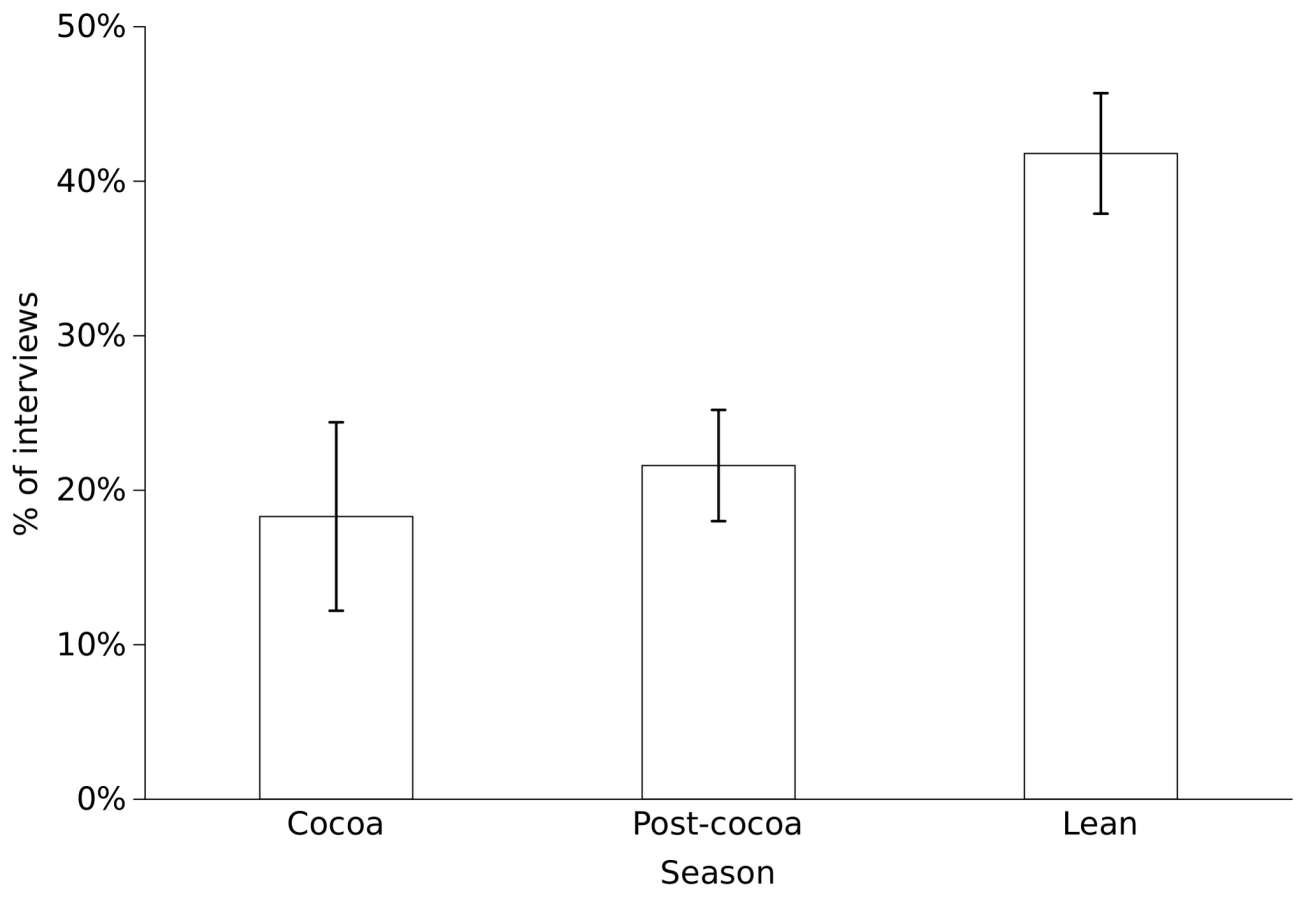
Seasonal variation in bushmeat sales. Percentage of interviews recording sale of part or all of bushmeat harvested within two weeks prior to the interview. Total sample size is 293 interviews across 50 households (including all interviews recording bushmeat within any two week recall period). Means and standard errors across households are shown.

In addition to bushmeat consumption reducing household expenditure, harvested bushmeat was more likely to be sold during the lean season (when cocoa income was lowest) than during the cocoa season, with 41.8% of interviews reporting the sale of harvested bushmeat during the lean season, compared to 18.3% and 21.6% during and post the cocoa harvest season respectively (Table 3, Figure 4). However, there was no substantial evidence for bushmeat sales being more important to FHHs or poorer households, or during the lean season.

**Table 3:** Results of binomial GLMM analysis testing the support for an effect of household wealth, seasonality and household gender on the likelihood of a household selling all or part of bushmeat harvested within last two weeks (N=293; No. households=50).

**Model**	**ΔAIC_i_**	**Akaike weight**
Season	0	0.58
Season + HH	1.9	0.23
Wealth + Season	3.6	0.10
Wealth + Season + HH	5.3	0.04
Season * HH	5.6	0.04
Wealth + Season * HH	8.9	0.01
Wealth * Season	9.8	<0.01
Wealth * Season + HH	11.5	<0.01
Null model	13.1	<0.01
Wealth * Season + Season * HH	15.0	<0.01
HH	15.0	<0.01
Wealth	16.4	<0.01
Wealth + HH	18.1	<0.01

## Discussion

The analyses indicate that among cocoa farmers in a faunally-depleted environment, the value of harvested bushmeat is relatively low and contributes little to household production, yet there is evidence that bushmeat is important during the agricultural lean season, providing a source of income or enabling households to save money and thereby provide a safety net function during a time of economic hardship. The following discussion focuses on evaluating our evidence for the safety net function of bushmeat.

The vast majority (89%) of households harvested bushmeat worth less than US$1.0/day, which is substantially less than has been reported from sites with abundant wildlife. For example, rural Gabonese hunters earned US$2.61/day [23] and trappers in the Central African Republic earned between US$1.3 and $1.9/day [45]. But our estimates are comparable to depleted environments in Côte d’Ivoire where hunters gained US$195 per annum [46], suggesting that the low bushmeat harvest and income gained from bushmeat in Wansampo is due to wildlife depletion.

However, this conclusion is challenged by other studies conducted in Ghana that reported substantially higher incomes from bushmeat sales. Natiamoa Baidu [13] interviewed hunters living in Ghana’s forest and savannah zone during a one week period of the agricultural lean season and derived a national average income from bushmeat sales of US$3.2/hunter/day. Possible reasons for the divergent results between Ntiamoa-Baidu’s study and the present study are that: (a) wildlife populations have declined in the ten years between her study and this study; (b) the short duration of her study during the agricultural lean season led to an unrepresentative estimation of seasonal bushmeat harvest; and (c) a focus on hunters who expended higher effort than was the case for hunters in this study (see 47 for estimates of hunting effort in Wansampo).

The importance of hunting effort is highlighted by a recent study of a Ghanaian bushmeat market. Professional hunters who invest the time to transport bushmeat from rural areas to town were shown to earn up to US$6 by selling a single grasscutter ( *Thryonomys* *swinderianus* ) [10], showing that bushmeat hunting within a landscape depleted of wildlife can be profitable provided hunters invest sufficient effort. In contrast, this study recorded bushmeat harvest and attempted harvest in only 20% of interviews, and when an animal was killed, this was often the result of an opportunistic harvest, e.g. checking farm traps while working on the farm [39,47].

So why was the bushmeat harvest so low in Wansampo? Ghanaian cocoa farmers have long been known as rational peasants [48], who adjust their livelihood activities depending on their opportunity cost and relative profitability. For example, Gyimah-Brempong [49], using longitudinal data to assess the supply-response function of Ghanaian cocoa production in relation to changes in the producer price of cocoa, and net income from cocoa sales relative to food crop production, concluded that farmers’ production decisions depended both on the price of cocoa and the profitability of food crop production relative to cocoa production. While we were unable to measure time allocation and the relative profitabilities of all livelihood activities in detail, it seems likely that the low bushmeat harvest was due not only to depleted wildlife, but also to the high opportunity costs of bushmeat hunting linked with high labour demands on cocoa farms.

Nevertheless, despite severe wildlife depletion and the low value of bushmeat harvest, this study found some increase in bushmeat offtake and use during the lean season, when households earned least cash income [39], suggesting that bushmeat may provide a buffer against the effects of income shortage and act as a safety net. Similar responses to seasonal income shortages have been reported among hunters [31,50], fishers [51] and NTFP gatherers in general [30].

It could be argued that seasonal variation in the bushmeat harvest was due to the timing of the planting season and farmers wanting to protect their newly planted food plants from crop damage [52]. This is unlikely to be an important reason in Wansampo, as most bushmeat was killed in forest rather than farmland [39,47]. Some farmers have been shown to increase hunting effort during periods of low labour requirements [53] and one could argue that cocoa farmers are busiest during the cocoa season, resulting in low bushmeat harvest at this time. However, published estimates of seasonal variation in labour demand are ambiguous, indicating a less distinct pattern. Okali [54] found that Ghanaian cocoa farmers spent more days per month in their farms during the lean season than the cocoa season due to the high labour demand for weeding. This is contrasted by recent evidence from a large scale cocoa farmer survey in Ghana, which found that the busiest months each year are August to November (overlapping both the lean season and cocoa season in this study) and the least busy months are January to May (primarily the post-cocoa season) [55]. Direct observations in Wansampo, albeit unquantified, suggest little difference in labour demands across seasons as farm-focused activities, e.g. weeding, planting and spraying, during the lean and post cocoa season shift to village-based activities, e.g. cocoa drying and negotiations, during the cocoa harvest seasons. Overall, there is no strong evidence to suggest that seasonal variation in the bushmeat harvest was due to variation in farm labour requirements.

Interestingly, the agricultural lean season, when the bushmeat harvest peaked, coincided with the main fishing season in Ghana [45], suggesting that it is not a reduction in the availability of fish that drives the seasonal increase in hunting activity in Wansampo (contrary to the pattern suggested for other regions of Ghana [29]). The lack of such an effect might reflect the relatively low consumption of fish in the study community and, when consumed, the predominant use of dried fish (79% of fish protein consumed) [39]. The consumption of such preserved fish might act to smooth supply across seasons [56].

Further evidence for the safety net function of bushmeat in this study is provided by the negative effect of consuming harvested bushmeat on meat/fish purchases and hence household expenditure. Increasing the bushmeat harvest frequency during the lean season enables households to consume bushmeat more often during this period of income shortage [39] and thereby save money when cash income is lowest. Similarly, Coad et al. [57] recorded a reduction in food purchases among rural Gabonese hunters as their bushmeat harvest increased, thereby freeing up money to spend on other items. Reducing cash expenditure is especially important during the agricultural lean season to provide money for essential services, such as hospital bills [31].

The value of harvested bushmeat on days when harvest occurred was constant across seasons, however, the sale of part or all of the daily bushmeat harvest was most likely during the lean season, thereby providing income during a time of cocoa income shortage and helping households to smoothen seasonal income fluctuations. This confirms previous studies assessing bushmeat hunting and NTFP harvest in general in relation to income shortage [25,30,31,50]. Hunters commonly sell more of their catch as their harvest increases [19,57], but this selling of a surplus does not seem to be the case in this study. The fact that on the one hand daily harvest value was constant across seasons but on the other hand the likelihood of selling and consuming harvested bushmeat were both highest during the lean season, strongly suggests that during this period of income shortage, hunters put greater weight on the use of bushmeat for income generation by selling part of the already small catch, while during the cocoa season they were more likely to consume the whole catch, as they had income from cocoa sales.

Overall, these findings substantiate earlier studies highlighting that bushmeat is an important safety net, whose primary importance lies in its availability when other livelihoods are temporarily unavailable or fail because of stochastic events. This view puts more emphasis on the timing than the magnitude of the harvest [5]. The seasonal safety net function of bushmeat therefore appears to be robust to reductions in the abundance of wildlife and availability of high value large-bodied animals.

While this study highlights a safety net function of bushmeat during the lean season, it also shows that poor households do not depend more on bushmeat than wealthier households. In fact, there was no evidence for any wealth differentiation related to patterns of bushmeat harvest and use. This contrasts strongly with a number of studies which concluded that wealth had a strong effect on bushmeat harvest, whereby the wealthiest households [58] or medium-wealth households [22] harvest most, and that bushmeat and NTFPs in general comprise the largest share of total income/production among the poorest households in a community [59].

What might explain this unexpected result? Failure to detect a difference between households of different wealth could have been caused by low variation in wealth among wealth categories [60]. However, there was little indication that this might have been the case in Wansampo as the wealthiest households gained ten times higher mean daily cocoa income than the poorest.

One explanation for the absence of a wealth effect on the bushmeat harvest in Wansampo might be found in the wider economic context. Cocoa farmers in Wansampo have relatively high incomes (US$3.2/capita/day in 2008 purchasing power parity, see 39) compared to other bushmeat studies conducted in more remote locations where there are few alternative income sources to bushmeat. Even the poorest households have access to alternative cash incomes and earn more than US$0.6/capita/day (in purchasing power parity). Further, communities showing wealth differentiation in harvest patterns usually have access to abundant wildlife resources [23,57], while in this study, wildlife resources are so limited that it restricts opportunities for major wealth related differences in hunting behaviour to arise between households. Gun hunting, frequently cited as a more efficient hunting technique and giving rise to high hunting incomes [58,61], was of minor importance in Wansampo and most animals were trapped or collected by hand [39]. Firstly, this indicates that wealth-related access restrictions may only apply to communities where wildlife is abundant enough to make the expensive technologies worthwhile. Secondly, our results support a number of NTFP studies conducted among farming communities with diversified livelihoods that found no relationship between natural resource harvest and household wealth [62,63], suggesting that poorer households are not necessarily the most resource dependent within communities and that socio-economic determinants of resource use are likely to be more complex, particularly once households gain access to a wider range of livelihood opportunities.

Although FHHs are generally perceived as more vulnerable than others [64], there was no evidence that they showed a greater reliance on bushmeat in this study. On the contrary, female-headed households harvested less bushmeat than MHHs, despite controlling for the confounding effects of household composition. Women were not prohibited from checking traps and were recorded harvesting bushmeat from traps in farms that were set by their spouse. They also gathered snails and small mammals that were encountered in farms and along forest roads. However, cultural norms prevented women from setting traps themselves and checking traps in forests where most bushmeat was harvested [39]. Hence, their role may be seen as a helper similar to that reported among Central African net hunters, where women cooperate with men in group hunts by driving animals into nets held by men [13,65]. For a FHH to gain regular access to trapped bushmeat throughout the year, it is therefore necessary to have at least one active male household member, which most FHHs did not have.

In contrast, gathering snails is an important livelihood activity during the rainy season and women feature prominently in this activity [66]. However, the rainy season in Ghana (March to early July) only partly overlaps with the lean season (June to September) and few snails were available during the present study period, possibly a result of naturally low abundance during this year [67]. Overall, our findings suggest that the labour limitations known to apply to FHHs [68] extend to the harvest of bushmeat, thereby limiting the potential of vulnerable FHHs to take full advantage of bushmeat’s safety net function. This is supported by consistently low levels of bushmeat production by FHHs, without the seasonal increase during the lean season seen in MHHs.

Because most bushmeat-livelihoods studies have been conducted in areas with high wildlife abundance and few alternative livelihood opportunities, little is known about the importance of depleted wildlife resources for rural households with access to a diverse range of income generating opportunities. This is despite the fact that wildlife depletion and the integration of households into the wider cash economy is progressing in rural Africa [69,70]. Our main finding is that farmers may continue to use bushmeat as a significant component of their strategy for coping with periodic income shortages, despite heavy depletion of wildlife populations and having access to other livelihood options. This outcome is important to both the development and conservation community. First it highlights that income fluctuations commonly experienced by African cash croppers pose a threat to their livelihoods and wildlife resources act as a safety net contributing to livelihood security. Second, conservation approaches focusing on restricting access to wildlife through hunting bans and law enforcement may compromise livelihood security, especially during the lean season, and as a consequence may find it difficult to ensure law enforcement that is already notoriously under-resourced.

While this remains the case, conservation and development goals will conflict, weakening the case for conservation interventions to limit the impact of hunting. With the main conflict potential being during the agricultural lean season, we suggest that this conflict of interests might best be tackled through collaboration of the conservation, development and agricultural sectors to first promote cross-sectoral understanding of seasonal livelihood patterns and second promote seasonal diversification and income smoothing of livelihoods by encouraging access to a balance of new and existing income sources both on- and off-farm. While this approach may not in itself reduce hunting pressure, we anticipate that it would reduce the need for resource users to harvest bushmeat, and so facilitate direct conservation interventions such as the protection of land from hunting.
